# Impact of frontline treatment approach on outcomes of myeloid blast phase CML

**DOI:** 10.1186/s13045-021-01106-1

**Published:** 2021-06-15

**Authors:** Kapil Saxena, Elias Jabbour, Ghayas Issa, Koji Sasaki, Farhad Ravandi, Abhishek Maiti, Naval Daver, Tapan Kadia, Courtney D. DiNardo, Marina Konopleva, Jorge E. Cortes, Musa Yilmaz, Kelly Chien, Sherry Pierce, Hagop Kantarjian, Nicholas J. Short

**Affiliations:** 1 Division of Cancer Medicine, MD Anderson Cancer Center, Houston, TX USA; 2 Department of Leukemia, The University of Texas MD Anderson Cancer Center, 1515 Holcombe Blvd, Unit 0428, Houston, TX 77030 USA; 3 Georgia Cancer Center, Augusta, GA USA

**Keywords:** CML, Blast phase, Chemotherapy, Hypomethylating agent, TKI

## Abstract

**Background:**

The natural course of untreated chronic myeloid leukemia (CML) is progression to an aggressive blast phase. Even in the current era of BCR-ABL1 tyrosine kinase inhibitors (TKIs), the outcomes of blast phase CML remain poor with no consensus frontline treatment approach.

**Methods:**

We retrospectively analyzed the response rates and survival outcomes of 104 consecutive patients with myeloid blast phase CML (CML-MBP) treated from 2000 to 2019 based on 4 different frontline treatment approaches: intensive chemotherapy (IC) + TKI (*n* = 20), hypomethylating agent (HMA) + TKI (*n* = 20), TKI alone (*n* = 56), or IC alone (*n* = 8). We also evaluated the impact of TKI selection and subsequent allogeneic stem cell transplant (ASCT) on patient outcomes.

**Results:**

Response rates were similar between patients treated with IC + TKI and HMA + TKI. Compared to treatment with TKI alone, treatment with IC/HMA + TKI resulted in a higher rate of complete remission (CR) or CR with incomplete count recovery (CRi) (57.5% vs 33.9%, *p* < 0.05), a higher complete cytogenetic response rate (45% vs 10.7%, *p* < 0.001), and more patients proceeding to ASCT (32.5% vs 10.7%, *p* < 0.01). With a median follow-up of 6.7 years, long-term outcomes were similar between the IC + TKI and HMA + TKI groups. Combination therapy with IC/HMA + TKI was superior to therapy with TKI alone, including when analysis was limited to those treated with a 2nd/3rd-generation TKI. When using a 2nd/3rd-generation TKI, IC/HMA + TKI led to lower 5-year cumulative incidence of relapse (CIR; 44% vs 86%, *p* < 0.05) and superior 5-year event-free survival (EFS; 28% vs 0%, *p* < 0.05) and overall survival (OS; 34% vs 8%, *p* = 0.23) compared to TKI alone. Among patients who received IC/HMA + TKI, EFS and OS was superior for patients who received a 2nd/3rd generation TKI compared to those who received imatinib-based therapy. In a landmark analysis, 5-year OS was higher for patients who proceeded to ASCT (58% vs 22%, *p* = 0.12).

**Conclusions:**

Compared to patients treated with TKI alone for CML-MBP, treatment with IC + TKI or HMA + TKI led to improved response rates, CIR, EFS, and OS, particularly for patients who received a 2nd/3rd-generation TKI. Combination therapy with IC + TKI or HMA + TKI, rather than a TKI alone, should be considered the optimal treatment strategy for patients with CML-MBP.

**Supplementary Information:**

The online version contains supplementary material available at 10.1186/s13045-021-01106-1.

## Introduction

Chronic myeloid leukemia (CML), which is characterized by the presence of the Philadelphia chromosome (Ph +) and the resultant *BCR-ABL1* fusion, occurs in different phases dependent on the absence or presence of certain clinical features, including cytogenetic clonal evolution, basophilia, and elevated blast percentage [1]. CML presents in a chronic phase (CP) in about 95% of patients [2]. Approximately 5% of patients present with an advanced phase of disease, either in an accelerated (AP) or blast phase (BP) [2]. The natural history of untreated or TKI-resistant CML is progression to BP (with or without an intervening AP) within 3–8 years from diagnosis of CML-CP, with a rapidly fatal disease course upon onset of BP [3–5].

BCR-ABL1 TKIs have remarkably altered the prognosis of CML, with patients in CP now having a normal lifespan compared to their age-matched peers and the prospect of a cure within reach for those who enter a durable treatment-free remission (TFR) [6, 7]. In the pivotal phase III IRIS study comparing imatinib to chemoimmunotherapy (cytarabine + IFNα), the 10-year rate of progression to an advanced phase was reduced from 12.8% with chemoimmunotherapy to 6.9% with imatinib [8]. The 10-year cumulative incidence of BP in the large imatinib-based CML-study IV cohort was 5.8%, and this rate has reduced even further with the use of second-generation TKIs (dasatinib, nilotinib, bosutinib) in the frontline setting [9–12].

Unlike CML-CP, patients who either present with de novo CML-BP or progress to BP from a previous CP/AP still have dismal outcomes, and the median OS remains less than 1 year after diagnosis of BP [13]. Approximately 70–80% of BP cases occur in myeloid blast phase (MBP) and 20–30% occur as a lymphoid blast phase (LBP) or with blasts of mixed lineage [1, 5]. Patients with LBP have better outcomes than those with MBP [14]. For CML-MBP, treatment approaches vary from the use of single-agent TKI to acute myeloid leukemia (AML)-like induction therapy + TKI, and there is no consensus frontline treatment recommendation [13, 15–17]. In order to clarify the optimal therapy for these patients, we retrospectively analyzed all patients receiving frontline treatment for CML-MBP over the past 20 years at our institution and assessed response rates, depth of response, and survival outcomes across different frontline therapies.

## Methods

### Study design and participants

This was a retrospective study including patients ≥ 18 years of age treated in the frontline setting for CML-MBP between 2000 and 2019. MBP was defined as the presence of ≥ 20% bone marrow (BM) myeloblasts with or without concurrent extramedullary (EM) disease per the World Health Organization 2017 criteria for CML-BP. Patients could have received prior therapy (including TKIs) for CML in CP/AP. Exclusion criteria were the presence of EM-only disease or any prior therapy for CML-BP aside from hydroxyurea. Patients were divided into four frontline treatment approaches: (1) intensive chemotherapy (IC) + TKI, (2) hypomethylating agent (HMA) + TKI, (3) TKI alone, or (4) IC alone. IC was defined as a regimen containing ≥ 1 g/m^2^ of cytarabine. All patients in the HMA + TKI group received decitabine. For certain specified analyses, groups 1 and 2 were combined into an “IC/HMA + TKI” group. TKI therapy was divided into 1st-generation (imatinib), 2nd-generation (dasatinib, nilotinib, bosutinib), or 3rd-generation (ponatinib) agents. The retrospective study was conducted at a single academic medical center (The University of Texas MD Anderson Cancer Center), approved by the Institutional Review Board, and conducted in accordance with the Declaration of Helsinki.

### Response and outcome definitions

Morphologic/hematologic responses were assessed per AML response criteria, as defined according to European LeukemiaNet consensus guidelines [18]. Patients who achieved a best response of complete remission (CR), complete remission with incomplete hematological recovery (CRi), or morphologic leukemia-free state (MLFS) were considered responders, and all others were considered nonresponders. Complete cytogenetic response (CCyR) was defined as no detectable Ph + chromosome by conventional karyotyping. Major molecular response (MMR; MR^3^) was defined as a *BCR-ABL1/ABL1* ratio > 0.01 and ≤ 0.1% on the International Scale (IS). MR^4^ was defined as a *BCR-ABL1/ABL1* ratio > 0.0032% and ≤ 0.01% on the International Scale (IS). MR^4.5^ was defined as a *BCR-ABL1/ABL1* ratio ≤ 0.0032% on the International Scale (IS). All molecular responses were assessed by quantitative real-time PCR analysis at the molecular diagnostics laboratory at MD Anderson Cancer Center and were converted to the IS.

Relapse was defined as ≥ 5% BM blasts, new extramedullary disease, or appearance of peripheral blasts unrelated to BM recovery after an initial objective response. Cumulative incidence of relapse (CIR) was calculated from time of initial response (CR, CRi, MLFS) to relapse, censored for death in morphologic remission or if the patient was alive at last follow-up. Event-free survival (EFS) was defined as the time from diagnosis to lack of response, morphologic relapse after initial morphologic response, or death from any cause. Overall survival (OS) was defined as the time from diagnosis to death from any cause. EFS and OS were censored if the patient was alive at last follow-up.

### Statistical methods

Patient characteristics were analyzed using descriptive statistics, and survival analyses were performed using Kaplan–Meier methodology. To compare two groups, Chi-Square test was performed for categorical variables, and *t*-test or Mann–Whitney test was performed for continuous variables. Log-rank (Mantel-Cox) test was performed to compare Kaplan–Meier survival curves. All statistical analyses were conducted using GraphPad Prism version 8.4.3.

## Results

### Patient characteristics and study cohort

Between January 2000 and April 2019, 104 patients received frontline treatment for CML-MBP at our institution and met our inclusion criteria. Twenty patients were treated with IC + TKI, 20 with HMA + TKI, 56 with TKI alone, and 8 with IC alone. Baseline patient characteristics of each group are shown in Table 1. Patients in the IC + TKI group were generally younger than patients in the 3 other groups (median age: 47 years vs. 57 years, respectively). Overall, 91 patients (87.5%) progressed from a prior CP/AP, and 67 (64.4%) had received at least one prior TKI for preceding CP/AP. Prior exposure to chemotherapy and/or HMA for CML CP/AP is detailed in Additional file 1: Table 1. Nine patients (8.6%) had concurrent EM disease in addition to BM involvement at the time of MBP diagnosis. The most common additional chromosomal abnormalities (ACAs) were + 8, extra Ph, and 3q26 rearrangements; T315I was the most common *ABL1* mutation (Additional file 1: Tables 2, 3).

**Table 1:** Baseline patient characteristics at time of CML-MBP treatment initiation

Characteristic	*N* (%); median [range]			
	IC + TKI (*N* = 20)	HMA + TKI (*N* = 20)	TKI (*N* = 56)	IC (*N* = 8)
Age, years	47 [29–83]	56 [37–89]	57 [21–79]	56 [27–74]
Race/ethnicity				
White, non-Hispanic	10 (50%)	14 (70%)	37 (66%)	5 (62.5%)
White, Hispanic	4 (20%)	1 (5%)	2 (3.6%)	0
Black	4 (20%)	4 (20%)	14 (25%)	3 (37.5%)
Other	0	0	1 (1.8%)	0
Not stated	2 (10%)	1 (5%)	2 (3.6%)	0
Initial CML presentation as de novo MBP	5 (25%)	5 (25%)	3 (5.3%)	0
Year of treatment initiation for MBP	2013 [2007–2018]	2013 [2003–2019]	2004 [2000–2012]	2003 [2000–2003]
Prior regimens for CML*	1 [0–3]	1 [0–4]	1.5 [0–5]	3 [0–4]
Prior TKI exposure	14 (70%)	14 (70%)	32 (60%)	7 (87.5%)
Changed TKI for MBP	10/14	7/14	30/32	N/A
BM blasts (%)	39 [21–87]	52 [24–91]	47 [20–87]	30 (20–60)
EM disease at diagnosis^	3/20 (15%)	0/20 (0%)	4/56 (7.1%)	2/8 (25%)
Additional clonal cytogenetic abnormalities	12 (60%)	15 (75%)	42 (75%)	6 (75%)
T315I mutation	3/20 (15%)	0/14 (0%)	1/23 (4.3%)	N/A
WBC (× 10^9^/L)	21.9 [3.1–259.3]	37.7 [1.0–156.6]	23.8 [0.7–363.7]	32.4 [2.4–319]
Platelet (× 10^9^/L)	127 [7–607]	75 [12–431]	82 [7–1128]	52 [21–2750]
Initial TKI for MBP				
Imatinib	0	7 (35%)	26 (48%)	N/A
Dasatinib	10 (50%)	11 (55%)	12 (21%)	N/A
Nilotinib	2 (10%)	1 (5%)	12 (21%)	N/A
Bosutinib	1 (5%)	0 (0%)	3 (5%)	N/A
Ponatinib	7 (35%)	1 (5%)	3 (5%)	N/A

*IC* intensive chemotherapy, *HMA* hypomethylating agent, *WBC* white blood cell count, *TKI* tyrosine kinase inhibitor, *MBP* myeloid blast phase, *BM* bone marrow, *EM* extramedullary disease

*Not including hydroxyurea

^ biopsy-confirmed

In the IC + TKI cohort, all patients received a 2nd/3rd-generation TKI as part of their initial CML-MBP therapy. In contrast, 65% and 52% of patients in the HMA + TKI and TKI alone groups, respectively, received a 2nd/3rd-generation TKI as their initial TKI for CML-MBP. Treatment regimens and year of therapy initiation are shown in Additional file 1: Table 4. The median year of MBP treatment initiation was 2013 for both IC + TKI and HMA + TKI compared to 2003 and 2004 for TKI alone or IC, respectively. These differences in therapies reflect a change in institutional practice around 2009 to preferentially use combination therapy with IC/HMA + TKI, rather than TKI alone, for patients with CML-MBP.

### Response rates

Morphologic, cytogenetic, and molecular responses rates are shown in Table 2. Patients treated with IC alone had the lowest response rate with only one objective response (CRi) among 8 IC-treated patients. Overall, responses rates were similar in patients treated with IC + TKI or HMA + TKI (CR/CRi rates 60% vs 55%, CCyR rates 40% vs 50%, MMR or deeper molecular response rates 29.4% vs 18.8%, respectively). Given similar responses between the IC + TKI and HMA + TKI groups, we combined these two groups for additional analyses (*n* = 40). Compared to treatment with TKI alone, combination therapy (IC/HMA + TKI) resulted in higher rates of CR/CRi (57.5% vs 33.9%, *p* < 0.05), CCyR (45% vs 10.7%, *p* < 0.001), and MMR or deeper molecular response (24.2% vs 4.3%, *p* < 0.01).

**Table 2:** Outcomes

Characteristic	N (%); Median [Range]			
	IC + TKI (*N* = 20)	HMA + TKI (*N* = 20)	TKI (*N* = 56)	IC (*N* = 8)
Best response				
CR	9 (45%)	6 (30%)	12 (21.4%)	0
CRi	3 (15%)	5 (25%)	7 (12.5%)	1 (12.5%)
MLFS	4 (20%)	3 (15%)	8 (14.3%)	0
PR	0	0	1 (1.8%)	0
No response	4 (20%)	6 (30%)	28 (50%)	7 (87.5%)
CR/CRi	12 (60%)	11 (55%)	19 (33.9%)	1 (12.5%)
ORR (CR/CRi/MLFS)	16 (80%)	14 (70%)	27 (48.2%)	1 (12.5%)
Complete cytogenetic remission	8 (40%)	10 (50%)	6 (10.7%)	0
Best molecular response^				
MMR	2/17 (11.7%)	1/16 (6.3%)	1/47 (2.1%)	0
MR^4^	2/17 (11.7%)	2/16 (12.5%)	0	0
MR^4.5^	1/17 (5.9%)	0	1/47 (2.1%)	0
Time to best response (months)	0.9 [0.7–6.9]	2.2 [0.8–5.5]	2.1 [0.6–16.3]	0.6 [0.6–0.6]
Proceeded to ASCT on this regimen	7 (35%)	6 (30%)	6* (10.7%)	1 (12.5%)
Median time to ASCT (months)	3.4 [1.5–7.9]	5.7 [2.9–8]	3.5 [2.5–5.7]	1.3 [1.3–1.3]
Median EFS (months)	5.2 [0.8–160.7]	5.0 [1.2–96.1]	4.8 [0.5–129.6]	2.2 [0.8–4.1]
Median RFS (months)	5.5 [0.5–159.8]	4.7 [0.6–93.5]	4.6 [0.2–127.7]	3.5 [3.5–3.5]
Median OS (months)	12.9 [0.8–160.7]	10.1 [1.2–96.1]	10.7 [0.5–244.3]	3.4 [0.8–48.9]
Relapse after initial objective response	6/16	6/14	17/27	0/1
EM relapse**	3/6 (50%)	1/6 (16.7%)	3/17 (17.6%)	0/1 (0%)
Early mortality				
30-day mortality	1 (5%)	0	1 (1.8%)	1 (12.5%)
60-day mortality	3 (15%)	2 (10%)	1 (1.8%)	3 (37.5%)
5-year rates				
CIR	51%	54%	80%	100%
EFS	27%	19%	5%	0%
OS	30%	28%	13%	0%

CR, complete remission; CRi, complete remission with incomplete count recovery; PR, partial remission; MLFS, morphologic leukemia-free state; ORR, overall response rate; ASCT, allogeneic stem cell transplantation; MMR, major molecular response; CMR, complete molecular response; CIR, cumulative incidence of relapse; EFS, event-free survival; RFS, relapse-free survival; OS, overall survival

^censored at time of SCT or first event, definitions below:

MMR (MR^3^): *BCR-ABL1* > 0.01% to ≤ 0.1% on the international scale (IS)

MR^4^: *BCR-ABL1* > 0.0032% to ≤ 0.01% on the IS

MR^4.5^: *BCR-ABL1* ≤ 0.0032% on the IS

*2 of the 6 patients in the TKI group went to ASCT with active disease

**Including central nervous system (CNS) relapse; one patient had CNS relapse in IC/TKI cohort, all other EM relapses were outside the CNS

### Relapse and survival outcomes

**Fig. 1:**
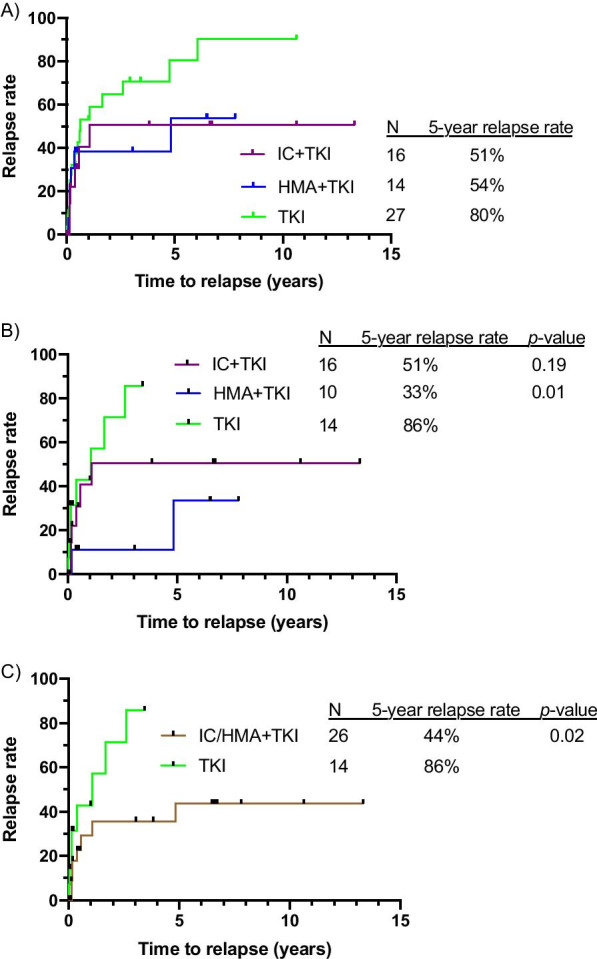
Cumulative incidence of relapse (CIR) based on a therapeutic approach including (**a**) any TKI or (**b** and **c**) only including regimens with a 2nd/3rd-generation TKI. *p*-values are between the indicated group and the TKI monotherapy group

Relapse and survival outcomes are shown in Table 2. Overall, the 5-year CIR rate was similar in patients who received IC + TKI or HMA + TKI, and the 5-year CIR rate was lower in patients treated with either IC + TKI or HMA + TKI compared to patients treated with TKI alone (Fig. 1a). The lower CIR with IC/HMA + TKI was observed in both the entire cohort and in the subgroup of patients treated with a 2nd/3rd-generation TKI (Fig. 1b). For patients treated with combination therapy (IC/HMA + TKI), the 5-year CIR rate was significantly lower compared to TKI monotherapy when a 2nd/3rd-generation TKI was used (44% vs 86%, *p* = 0.02) (Fig. 1c).

**Fig. 2:**
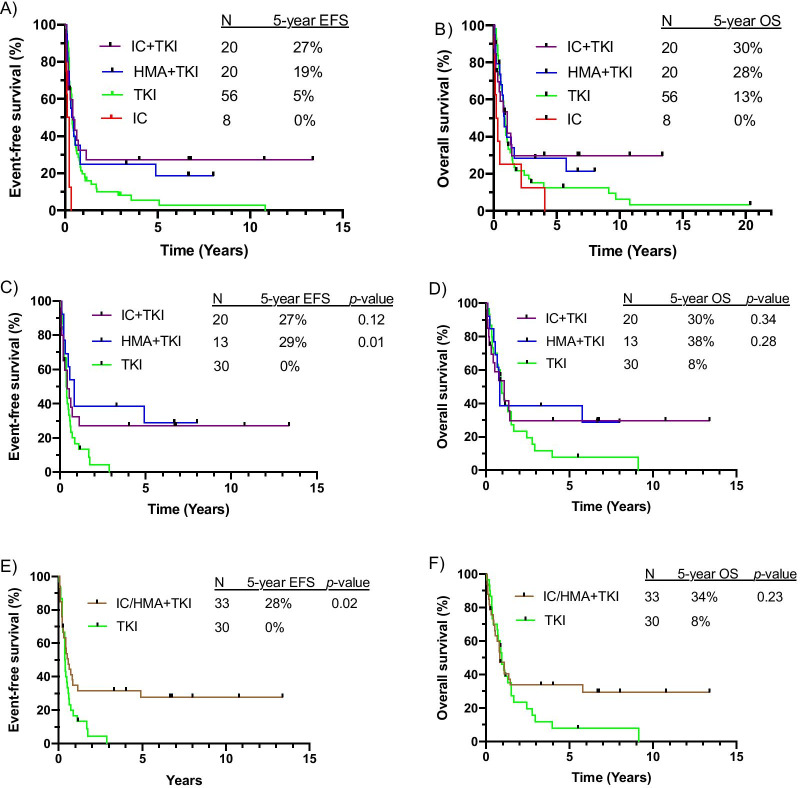
Outcomes based on any therapeutic approach for (**a**) event-free survival (EFS) and (**b**) overall survival (OS). Outcomes for patients who received 2nd/3rd-generation TKI for (**c**) EFS and (**d**) OS. Outcomes for patients who received combination therapy versus TKI monotherapy for (**e**) EFS and (**f**) OS). *p*-values are between the indicated group and the TKI monotherapy group

Though median EFS and OS were similar between the IC + TKI, HMA + TKI, and TKI alone groups (Table 2), long-term survival outcomes differed among the treatment groups. With median follow-up of 6.7 years, 5-year EFS and OS was superior for patients treated with combination therapy compared to those treated with TKI alone (Fig. 2a, b). The superior outcomes with IC/HMA + TKI were observed despite a higher 60-day mortality for patients treated with combination therapy compared to TKI alone (12.5% vs 1.8%, *p* < 0.05, Table 2; cause of 60-day mortality listed in Additional file 1: Table 5). When limited to patients treated with a 2nd/3rd-generation TKI, 5-year EFS was 0% for those treated with TKI alone, 27% with IC + TKI, and 29% with HMA + TKI (Fig. 2c). 5-year OS was 8% for those treated with TKI alone, 30% with IC + TKI, and 38% with HMA + TKI, (Fig. 2d). Among patients who received a 2nd/3rd-generation TKI, combination therapy with IC/HMA + TKI resulted in improved 5-year EFS (28% vs 0%, *p* < 0.05; Fig. 2e) and 5-year OS (34% vs 8%, *p* = 0.23; Fig. 2f) compared to treatment with TKI alone.

Lastly, the effect of ACAs on clinical outcome was examined. Given that all 4 groups had a similar number of patients with at least one ACA (60% in IC + TKI, 75% in the other 3 groups), patients with at least 1 ACA (*n* = 75) were compared to patients without an ACA (*n* = 29). As has been described previously, the presence of ACAs conferred worse EFS and OS compared to no ACAs (Additional file 1: Fig. 1) [19].

### Impact of TKI received on response and survival outcomes

**Fig. 3:**
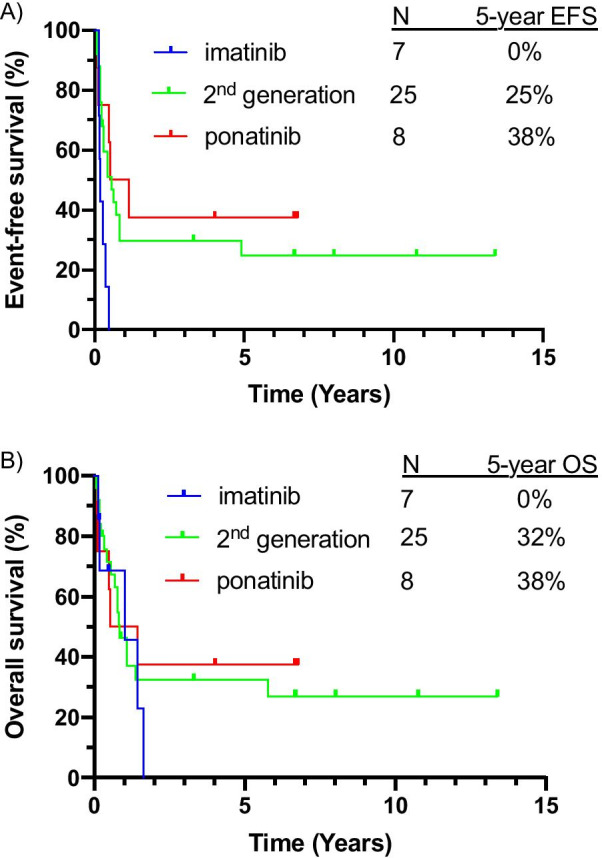
Outcomes stratified by TKI received for patients treated with combination therapy plus a TKI for (**a**) event-free survival and (**b**) overall survival

Given the increasing use of a 2nd/3rd-generation TKI in the treatment of CML-MBP as opposed to an imatinib-based regimen, we assessed outcomes for patients treated with IC/HMA + TKI based on TKI generation. Of the 40 patients treated with IC/HMA + TKI, 7 were treated with an imatinib-based regimen, 25 with a 2nd-generation TKI-based regimen, and 8 with a ponatinib-based regimen. Four out of 57 tested patients (7%) had a T315I mutation at the time of CML-MBP diagnosis (Table 1), and 1/8 patients treated with ponatinib had a detectable T315I mutation. Response rates and survival outcomes by TKI are shown in Table 3. Imatinib-based combination therapy was associated with very poor outcomes, with 5-year EFS and OS rates of 0% compared to 5-year EFS and OS rates of 25% and 32%, respectively, with a 2nd-generation TKI-containing regimen, and 5-year EFS and OS rates of 38% and 38%, respectively, with a ponatinib-based regimen (Fig. 3a, b).

**Table 3:** Outcomes with IC/HMA + TKI based on TKI generation

Outcome	*N* (%)		
	IC/HMA + imatinib (*N* = 7)	IC/HMA + 2nd generation TKI (*N* = 25)	IC/HMA + ponatinib (*N* = 8)
CR	1 (14.3%)	11 (44%)	3 (37.5%)
CR/CRi	2 (28.6%)	16 (64%)	5 (62.5%)
CR/CRi/MLFS	4 (57.1%)	19 (76%)	7 (87.5%)
5-year CIR	100%	45%	38%
5-year EFS	0%	25%	38%
5-year OS	0%	32%	38%

CR, complete remission; CRi, complete remission with incomplete count recovery; MLFS, morphologic leukemia-free state;

CIR, cumulative incidence of relapse; EFS, event-free survival; OS, overall survival

### Impact of ASCT on survival outcomes

**Fig. 4:**
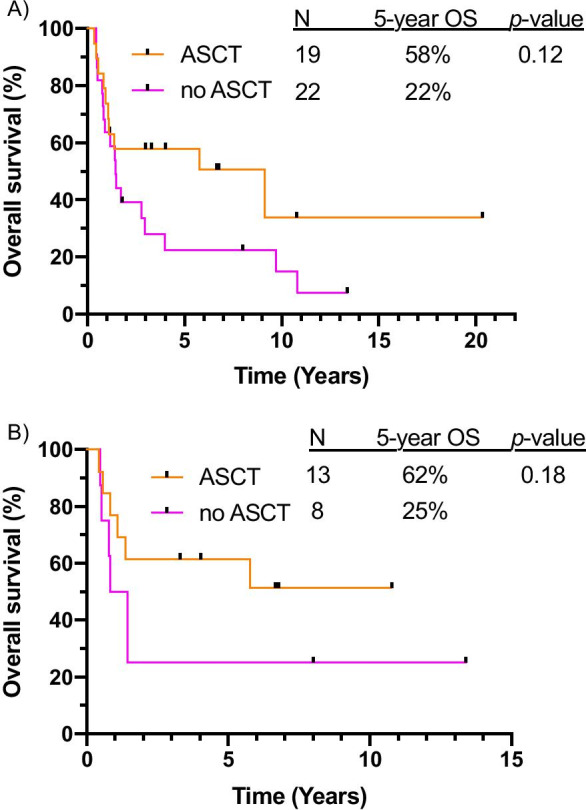
Landmark overall survival analysis for transplanted versus non-transplanted patients, including (**a**) all patients in the cohort and (**b**) only those who received combination therapy plus a TKI

Treatment with combination therapy (IC/HMA + TKI) led to more patients proceeding to ASCT compared to TKI alone (32.5% vs 10.7%, *p* < 0.01). Median time to ASCT was 4.7 months in the entire cohort of patients and 4.9 months for patients treated with IC/HMA + TKI. Landmark survival analysis was performed on patients with a morphologic response (i.e. CR, CRi, or MLFS) who were alive and event-free at the landmark time point. Those who underwent ASCT (*n* = 19) had superior OS compared to those who did not (*n* = 22), with 5-year OS rates of 58% vs. 22% (*p* = 0.12) (Fig. 4a). Similar benefit to ASCT was seen when only including patients treated with IC/HMA + TKI (Fig. 4b).

Notably, 4 patients survived > 5 years despite not undergoing ASCT. One patient was treated with IC + TKI followed by maintenance TKI, 1 patient with HMA + TKI followed by maintenance TKI, and 2 patients with imatinib alone. Of the 4 patients, only one relapsed. This patient was treated with imatinib initially followed by different single-agent TKI regimens due to treatment intolerance and ultimately relapsed 6 years after initial MBP diagnosis.

## Discussion

Outcomes for CML-CP have improved considerably in the TKI era [13]. However, the occurrence of BP remains a challenging clinical scenario and for many patients is an end-stage event [6]. Due to its rarity, treatment is guided primarily by retrospective series, small single-arm clinical trials, and expert opinion. In this study, we identified 104 patients with CML-MBP over a 20-year period treated with different therapeutic approaches in the frontline setting. To our knowledge, this is the largest cohort of patients with CML-MBP to include data on response rates, relapse incidence, and survival based on therapeutic approach. We found that therapy with IC/HMA + TKI in comparison to TKI alone yields higher response rates, lower risk of relapse, and improved 5-year EFS/OS, suggesting that combination therapy with IC or HMA plus a TKI should be consider the optimal upfront therapy for these patients, rather than TKI alone.

Although there are several published algorithms on how to approach CML-MBP, there is no formal category 1 recommendation in clinical guidelines. Trial enrollment, AML-like induction therapy + TKI, or TKI alone are all considered acceptable treatment options [17]. The incorporation of a TKI is crucial, as the BCR-ABL1 fusion kinase remains a significant leukemic driver in patients with CML-BP. For historical comparison to demonstrate this point, we included patients in our study treated with IC alone. While the number of patients in our cohort who received IC alone is small, it is evident that IC alone is inadequate therapy, with a response rate of 12.5% and 5-year EFS and OS rates of 0%. The utilization of TKI monotherapy instead of IC improved outcomes for patients, however long-term outcomes remained poor with a 5-year EFS rate of 5% and OS rate of 13%. While the subsequent use of combination regimens (IC/HMA + TKI) did not significantly improve median EFS/OS compared to TKI, there was a clear benefit in the proportion of patients achieving long-term survival.

The utilization of combination therapy for CML-MBP may have yielded improved responses compared to TKI alone because progression to MBP, with or without the development of a resistant *ABL1* mutation, suggests development of leukemogenic events independent of the BCR-ABL1 kinase [5, 20]. The biology of BP is much less understood compared to CP. Patients with BP may have mutations in genes commonly associated with AML, such as *ASXL1* and *RUNX1*, as well as ACAs that confer a negative prognosis in AML, such as − 7 and inv(3) [19, 21, 22]. These mutations and ACAs along with ongoing constitutive BCR-ABL1 kinase activity and additional cellular changes yield a complex disease biology in which both proliferative and anti-apoptotic pathways have been implicated, including but not limited to those mediated by Wnt/β-catenin, MYC, and BCL-2/BCL-xL [5, 23]. The progression to MBP therefore appears to create a more heterogenous disease than CML-CP, and targeting a single driver pathway may be insufficient. Given the proliferation of myeloblasts and immature progenitor cells, aspects of the disease clinically appear to mimic AML more so than CML-CP. Thus, the use of an acute leukemia chemotherapeutic regimen + TKI may be efficacious by affecting multiple cell survival/death mechanisms rather than one primary target, as has been shown for patients with Ph + acute lymphoblastic leukemia (ALL) treated with TKI-based combination regimens [24].

Patients treated with combination therapy had improved long-term survival in our cohort, and it appears that the etiology of this benefit primarily arose from a higher rate of patients proceeding to ASCT (32.5% with IC/HMA + TKI vs. 10.7% with TKI alone). Among patients who had a clinical response and underwent ASCT, 5-year OS was 58% for all patients (62% for those treated with IC/HMA + TKI). Prior to the TKI era, ASCT was the only curative option for patients with any phase of CML, and it remains the therapeutic goal for patients with CML-BP [25]. In AML, Ph + ALL, and CML-CP, outcomes post-ASCT are influenced by the pre-transplant depth of response [26–28]. TKI therapy alone resulted in a CR/CRi rate of 33.9% and CCyR rate of 10.7%. In contrast, combination therapy produced a CR/CRi in 57.5% of patients and CCyR in 45% of patients. MMR or deeper molecular response rates were relatively low with combination therapy (29.4% with IC + TKI and 18.8% with HMA + TKI) but were still higher than the 4.3% rate with TKI alone. Thus, it is possible that the improved outcomes associated with IC/HMA + TKI were due to a higher likelihood of reducing leukemia burden pre-transplant, thereby permitting more patients to proceed to the potentially curative option of ASCT.

IC + TKI and HMA + TKI have been assessed separately in small, early phase clinical trials and retrospective studies [29–34]. However, to the best of our knowledge they have not been previously compared to each other within the same cohort. Though IC + TKI and HMA + TKI produced similar response rates and survival outcomes in this cohort, it is notable that patients in the IC + TKI group were younger, only received a 2nd/3rd-generation TKI, and were more likely to receive ponatinib compared to patients treated with HMA + TKI. Though 60-day mortality was similar between both groups, HMAs are typically better tolerated with fewer toxicities compared to most IC regimens for AML. With regards to which TKI to use in combination therapy, no patient treated with an imatinib-based combination regimen survived past 2 years, further supporting the use of later-generation TKIs in this context. Assessing for differences between patients treated with ponatinib or a 2nd-generation TKI is limited by the small number of patients treated with ponatinib (*n* = 8). However, 5-year EFS and OS rates were similar or slightly higher with the use of ponatinib compared to 2nd-generation TKIs, which parallels findings in Ph + ALL where the use of ponatinib as the frontline TKI has demonstrated significant clinical efficacy even in the absence of a T315I mutation [35, 36]. Ongoing studies are evaluating lower-intensity therapy (HMA + venetoclax) combined with ponatinib for patients with Ph + myeloid malignancies, including CML-MBP (NCT04188405), which may further improve outcomes for this disease. Retrospective and prospective studies also support the use of venetoclax-based combination regimens in CML-BP and advanced Ph + leukemias [37, 38].

Our study is limited primarily due to its retrospective, nonrandomized, single-center methodology. Thus, we were unable to capture certain types of clinical information that are typically gathered for prospective clinical studies and may have influenced treatment outcomes, such as patient performance status, physician rationale for treatment choice, and adverse events. Due to the declining incidence of CML-MBP, prospective randomized trials are unlikely to occur and, if so, will likely require significant time to accrue. We aimed to minimize the potential biases inherent in a retrospective cohort analysis by having relatively strict inclusion criteria and focusing on patients receiving their first therapy for CML-MBP and excluding patients with EM-only disease. We focused on patients who received frontline treatment for CML-MBP in order to minimize the variability associated with including patients within the same analysis who had no prior BP treatment with those who had relapsed/refractory disease to one or more prior BP regimens. For patients with EM-only disease, responses as assessed by imaging rather than typical CML or AML hematologic and molecular criteria would have complicated our response assessment, particularly in a retrospective analysis where imaging modalities were not consistently used.

## Conclusion

In summary, our study supports the use of combination therapy with IC + TKI or HMA + TKI rather than TKI monotherapy for patients with CML-MBP. IC + TKI and HMA + TKI produced similar results, with long-term survival of approximately 30%, and superior outcomes in those patients who were able to proceed to ASCT. In particular, the combination of HMA + 2nd/3rd-generation TKI is highly effective, despite being less intensive than an IC-based approach. These data provide a valuable benchmark for future prospective studies evaluating novel therapies for patients with CML-MBP.

## Supplementary Information

**Additional file 1**. Supplementary Tables 1-5 and Supplementary Figure 1.

## Data Availability

All data generated and analyzed during this study are included in this published article and the supplementary files.
