# Supplementary material for: Impact of frontline treatment approach on outcomes of myeloid blast phase CML
Source: J Hematol Oncol. 2021 Jun 15;14:94. doi: 10.1186/s13045-021-01106-1 (PMC8204504; doi:10.1186/s13045-021-01106-1)
Supplement: Supplementary file 1 — Additional file 1. Supplementary Tables 1-5 and Supplementary Figure 1. [file 13045_2021_1106_MOESM1_ESM.docx]

Impact of Frontline Treatment Approach on Outcomes of Myeloid Blast Phase CML

Supplementary Information

**Supplementary Table 1.** Patients with exposure to HMA or chemotherapy for CML CP/AP

**Supplementary Table 2.** Additional chromosomal abnormalities (ACAs)

**Supplementary Table 3.** *ABL1* mutations at the time of MBP diagnosis

**Supplementary Table 4.** Treatment regimen by year

**Supplementary Table 5.** 60-day mortality

**Supplementary Figure 1.** Survival outcomes in patients with and without ACA(s)

**Supplementary Table 1.** Patients with exposure to HMA or chemotherapy for CML CP/AP

| **Group** | **IC + TKI**  **(N=20)** | **HMA + TKI (N=20)** | **TKI**  **(N=56)** | **IC**  **(N=8)** |
| --- | --- | --- | --- | --- |
| Patients with prior exposure to chemotherapy and/or HMA for CML CP/AP | 3 | 3 | 12 | 6 |
| Intensive chemotherapy | 1 | 1 | 1 | 0 |
| Non-intensive chemotherapy* | 0 | 2 | 9^#^ | 3^#^ |
| HMA | 2 | 0 | 3^#^ | 4^#^ |

* low-dose subcutaneous cytarabine, omacetaxine (homoharringtonine), oral busulfan

^#^ 1 patient in the TKI group and 1 patient in the the IC group were exposed to both an HMA and non-intensive chemotherapy at separate times and thus are counted under both “non-intensive chemotherapy” and “HMA”

**Supplementary Table 2.** Additional chromosomal abnormalities (ACAs) at the time of MBP diagnosis

| **ACA** | **Frequency among 75 patients with ACA(s)** |
| --- | --- |
| +8 | 20 (26.7%) |
| extra Ph* | 15 (20%) |
| 3q26 rearrangement^#^ | 14 (18.7%) |
| i(17q) | 10 (13.3%) |
| del7/7q | 4 (5.3%) |
| +21 | 4 (5.3%) |
| -Y | 4 (5.3%) |
| del5/5q | 3 (4%) |
| inv(16) | 3 (4%) |
| >2 ACAs | 34 (45.3%) |

- shown are abnormalities present in > 3 patients

- patients with more than one abnormality may be counted in more than one group if both abnormalities are included above

* extra Ph as previously described (reference 19) = der(22)t(9;22), der(22)idic(22)(q11.2)t(9;22), idic(22)(p11.2)t(9;22), ider(22)(q10)t(9;22)

^#^ 3q26 rearrangement as previously described (reference 19) = inv(3)(q21q26), t(3;3)(q21q26), t(3;21)(q26;q22)

**Supplementary Table 3.** *ABL1* mutations at the time of MBP diagnosis

| ***ABL1* mutation** | **Frequency among 57 patients with *ABL1* sequencing information** |
| --- | --- |
| T315I | 4 (7%) |
| F317L | 2 (3.5%) |
| M351T | 1 (1.8%) |
| M244V and M351T | 1 (1.8%) |
| Y253H | 1 (1.8%) |
| V299L | 1 (1.8%) |
| G706S | 1 (1.8%) |
| E355G | 1 (1.8%) |
| E292V | 1 (1.8%) |
| E255K | 1 (1.8%) |

**Supplementary Table 4.** Treatment regimen by year

| **Group** | **Backbone regimen** | **TKI** | **Start date (year)** |
| --- | --- | --- | --- |
| TKI | none | imatinib | 2000 |
| TKI | none | imatinib | 2000 |
| TKI | none | imatinib | 2000 |
| TKI | none | imatinib | 2000 |
| TKI | none | imatinib | 2000 |
| IC | FA | none | 2000 |
| TKI | none | imatinib | 2000 |
| TKI | none | imatinib | 2000 |
| TKI | none | imatinib | 2000 |
| TKI | none | imatinib | 2000 |
| TKI | none | imatinib | 2000 |
| TKI | none | imatinib | 2000 |
| TKI | none | imatinib | 2000 |
| TKI | none | imatinib | 2000 |
| TKI | none | imatinib | 2000 |
| TKI | none | imatinib | 2000 |
| TKI | none | imatinib | 2000 |
| TKI | none | imatinib | 2001 |
| TKI | none | imatinib | 2001 |
| TKI | none | imatinib | 2001 |
| IC | AraC + topotecan | none | 2001 |
| TKI | none | imatinib | 2001 |
| TKI | none | imatinib | 2001 |
| TKI | none | imatinib | 2001 |
| TKI | none | imatinib | 2001 |
| TKI | none | imatinib | 2002 |
| TKI | none | imatinib | 2002 |
| IC | clofarabine + AraC | none | 2003 |
| HMA+TKI | decitabine | imatinib | 2003 |
| IC | IA | none | 2003 |
| HMA+TKI | decitabine | imatinib | 2003 |
| TKI | none | imatinib | 2003 |
| IC | IA | none | 2003 |
| IC | IA | none | 2003 |
| IC | IA | none | 2003 |
| HMA+TKI | decitabine | imatinib | 2003 |
| HMA+TKI | decitabine | imatinib | 2003 |
| IC | IA | none | 2003 |
| HMA+TKI | decitabine | imatinib | 2003 |
| HMA+TKI | decitabine | imatinib | 2004 |
| TKI | none | nilotinib | 2004 |
| TKI | none | dasatinib | 2004 |
| TKI | none | dasatinib | 2004 |
| TKI | none | dasatinib | 2004 |
| TKI | none | dasatinib | 2004 |
| TKI | none | nilotinib | 2004 |
| TKI | none | dasatinib | 2004 |
| TKI | none | dasatinib | 2004 |
| TKI | none | nilotinib | 2004 |
| TKI | none | dasatinib | 2004 |
| TKI | none | nilotinib | 2004 |
| TKI | none | dasatinib | 2005 |
| TKI | none | nilotinib | 2005 |
| TKI | none | nilotinib | 2005 |
| HMA+TKI | decitabine | imatinib | 2005 |
| TKI | none | dasatinib | 2005 |
| TKI | none | dasatinib | 2005 |
| TKI | none | dasatinib | 2005 |
| TKI | none | nilotinib | 2005 |
| TKI | none | nilotinib | 2005 |
| TKI | none | nilotinib | 2005 |
| TKI | none | dasatinib | 2006 |
| TKI | none | nilotinib | 2006 |
| TKI | none | nilotinib | 2006 |
| TKI | none | bosutinib | 2006 |
| IC+TKI | IA | dasatinib | 2007 |
| TKI | none | bosutinib | 2007 |
| TKI | none | bosutinib | 2007 |
| TKI | none | nilotinib | 2008 |
| IC+TKI | IA | nilotinib | 2009 |
| IC+TKI | FA | dasatinib | 2009 |
| IC+TKI | IA | nilotinib | 2010 |
| IC+TKI | FA | dasatinib | 2010 |
| IC+TKI | IA | dasatinib | 2011 |
| IC+TKI | FA | dasatinib | 2011 |
| TKI | none | ponatinib | 2012 |
| TKI | none | ponatinib | 2012 |
| TKI | none | ponatinib | 2012 |
| HMA+TKI | decitabine | dasatinib | 2012 |
| HMA+TKI | decitabine | nilotinib | 2012 |
| IC+TKI | IA | dasatinib | 2012 |
| HMA+TKI | decitabine | dasatinib | 2013 |
| HMA+TKI | decitabine | dasatinib | 2013 |
| IC+TKI | FA | ponatinib | 2013 |
| IC+TKI | CIA | ponatinib | 2013 |
| HMA+TKI | decitabine | dasatinib | 2013 |
| HMA+TKI | decitabine | dasatinib | 2014 |
| IC+TKI | FLAG-Ida | ponatinib | 2014 |
| HMA+TKI | decitabine | dasatinib | 2014 |
| HMA+TKI | decitabine | ponatinib | 2014 |
| HMA+TKI | decitabine | dasatinib | 2015 |
| HMA+TKI | decitabine | dasatinib | 2015 |
| IC+TKI | CLIA | ponatinib | 2016 |
| HMA+TKI | decitabine | dasatinib | 2016 |
| IC+TKI | FIA | dasatinib | 2016 |
| IC+TKI | CLIA | dasatinib | 2016 |
| IC+TKI | CLIA | ponatinib | 2016 |
| IC+TKI | FA | bosutinib | 2016 |
| IC+TKI | FIA | dasatinib | 2017 |
| IC+TKI | CLIA | dasatinib | 2018 |
| IC+TKI | CLIA | ponatinib | 2018 |
| HMA+TKI | decitabine | dasatinib | 2018 |
| IC+TKI | CLIA | ponatinib | 2018 |
| HMA+TKI | decitabine | dasatinib | 2019 |

AraC = cytarabine; FA = fludarabine/AraC; IA = idarubicin/AraC; FA = fludarabine/AraC; CIA = clofarabine/idarubicin/AraC;

CLIA = cladribine/idarubicin/AraC; FLAG-Ida = fludarabine/AraC/GCSF/idarubicin

**Supplementary Table 5.** 60-day mortality

| **Patient Group** | **Overall survival (months)** | **Cause of death** | **Disease status at time of death** |
| --- | --- | --- | --- |
| IC+TKI | 1.1 | seizure versus stroke | too early to evaluate |
| IC+TKI | 0.8 | unknown (out of hospital) | too early to evaluate |
| IC+TKI | 1.2 | pulmonary hemorrhage | MLFS |
| HMA+TKI | 1.7 | pneumonia, renal failure | no response, active disease |
| HMA+TKI | 1.2 | pneumonia | too early to evaluate |
| TKI | 0.5 | unknown (out of hospital) | too early to evaluate |
| IC | 1.5 | unknown (out of hospital) | no response, active disease |
| IC | 1.3 | transitioned to hospice | too early to evaluate |
| IC | 0.8 | pneumonia, multiorgan failure | too early to evaluate |

MLFS = morphologic leukemia-free state;

**Supplementary Figure 1.** Survival outcomes in patients with and without ACA(s)

A)

B)

Outcomes among total population of 104 patients based on the presence or absence of additional chromosomal abnormalities for (A) event-free survival (EFS) and (B) overall survival (OS).
